# Stability of lutein encapsulated whey protein nano-emulsion during storage

**DOI:** 10.1371/journal.pone.0192511

**Published:** 2018-02-07

**Authors:** Changhui Zhao, Xue Shen, Mingruo Guo

**Affiliations:** 1 College of Food Science and Engineering, Jilin University, Changchun, China; 2 Key Laboratory of Zoonosis, Ministry of Education, College of Veterinary Medicine, Jilin University, Changchun, China; 3 Department of food science, Northeast Agriculture University, Harbin, China; 4 Department of Nutrition and Food Sciences, University of Vermont, Burlington, Vermont, United States of America; Fudan University, CHINA

## Abstract

Lutein is a hydrophobic carotenoid that has multiple health functions. However, the application of lutein is limited due to its poor solubility in water and instability under certain conditions during storage. Hereby we generated lutein loaded nano-emulsions using whey protein isolate (WPI) or polymerized whey protein isolate (PWP) with assistance of high intensity ultrasound and evaluate their stability during storage at different conditions. We measured the particle size, zeta-potential, physical stability and lutein content change. Results showed that the PWP based nano-emulsion system was not stable in the tested Oil/Water/Ethanol system indicated by the appearance of stratification within only one week. The WPI based nano-emulsion system showed stable physiochemical stability during the storage at 4°C. The lutein content of the system was reduced by only 4% after four weeks storage at 4°C. In conclusion, our whey protein based nano-emulsion system provides a promising strategy for encapsulation of lutein or other hydrophobic bioactive molecules to expand their applications.

## Introduction

Lutein is a hydrophobic carotenoid abundantly present in dark green leafy vegetables. They are commercially extracted from the Marigold flowers (*Tagetes erecta*) for production of orange colorings for the food and beverage industries and dietary supplements. Carotenoids cannot be synthesized *de novo* in body [1, 2] and have to be obtained from the diet. Free or unesterified lutein obtained from the foods is transported and stored in the liver or in tegumentary sites as di-esterified lutein [3–5]. Supplementation with lutein can lead to an increment of lutein concentration in human serum and increase the optical density in the retina [6]. Because of the presence of lutein in the eye tissue [7], lutein supplementation can help reduce the risk of some age-related eye diseases including cataract and macular degeneration [8, 9]. In addition, high intake of lutein can reduce the risk of cardiovascular diseases [10, 11], cancers [12–14], as well as other diseases like atherosclerosis [15]. Since lutein is not rich in our common diet, adding certain carotenoids into our foods is therefore recommended [16].

Unfortunately, the application of lutein is limited due to its poor solubility in aqueous phase and instability under certain conditions like oxygens, heat, light or humidity [17–19]. Incorporation of lutein into a protective matrix is a promising means to address these problems, for example, micro-capsulation of lutein with porous starch and gelatin mixture using spray drying [20], encapsulation of lutein with hydroxypropylmethyl cellulose phthalate by supercritical antisolvent method [21], formation of lipid nanoparticles of lutein for dermal delivery by high pressure homogenization [22] and encapsulation of lutein into emulsions using different emulsifiers like polyvinylpyrrolidone [23], soy protein isolate [24], whey protein products [25], etc.. Selecting proper packaging materials is important for the stability and bioaccessibility of the bioactive compounds.

The milk protein is an important additive in many products. The whey protein and casein are the most protein components in milk, both of which are good emulsifiers to prepare stable lutein-loaded emulsions [25, 26]. Whey protein isolate (WPI) is a commercially available protein product that contains over 90% of protein. WPI is less efficient than casein to stabilize lutein [27], but use of WPI is more efficiently to protect the lutein from color degradation during storage [28]. Additionally, whey protein is costly and can improve the nutritional values [29, 30]. The health benefits of whey protein have extended beyond its basic protein nutrition for adults including adiposity control, promotion of muscle protein synthesis, enhancement of immune function and anti-oxidant activity [31]. Frede *et*. *al*. generated β-lactoglobulin and whey protein hydrolysate based lutein loaded emulsions with the particle size in the range from 250 to 300 nm. This system is promising as it can protect lutein from degradation within 46 days tested [25]. Our recent research showed that the emulsifying ability of WPI was increased by thermal treatment, which resulted in formation of whey protein soluble aggregates called polymerized whey protein (PWP) [32]. However, the stability of the emulsion system with commercial WPI or PWP as the emulsifiers is not studied at different storage conditions.

High intensity ultrasound is considered to be a promising technique that is relatively economical and easy to operate. Several studies have shown that ultrasound treatment can change the gelation properties of different food proteins [33–36], including whey protein [37]. Therefore, the objective of the current research was to generate a WPI based lutein loaded nano-emulsion system using ultra intensity ultrasound and evaluate its stability at different storage conditions.

## Materials and methods

### Materials and reagents

Whey protein isolate (93.1% of protein: α-lactalbumin: β-lactoglobulin = 3.5:1, 0.36% of fat, 4.8% of moisture, 1.6% of ash and 0.7% of lactose) was purchased from Fonterra (Auckland, New Zealand). Lutein (99.0%) was from Kemai, China. All other reagents were of analytical grade.

### Preparation of lutein-loaded emulsions

The lutein-whey protein nano-emulsion (20%, v/v; oil-in-water) was prepared using the method from Frede *et*. *al*. with slight modifications [25]. To form a pre-emulsion 1 ml of the oil phase and 4 ml of the aqueous phase were mixed and stirred. The pre-emulsion was homogenized using high intensity ultrasound for 5 min in an ice bath at the amplitude of 40%. The oil phase contained 2.5mM or other specified amount of lutein dispersed in Ethanol/MCT-oil (50/50, v/v). The aqueous phase was 5% or other specified amount of WPI solution or polymerized WPI (PWP), which was prepared as previously reported [38]. The emulsions were stored at 4°C, 25°C and 37°C for four weeks.

### Particle size and zeta-potential measurements

The particle size and the zeta-potential (ζ, mV) was determined using the Zetasizer Nano ZS 90 (Malvern Instruments, UK) as previously reported [37, 39]. Polydispersity index (PDI) was also measured reflecting the width of particle size distributions.

ζ was calculated based on the Henry equation:

$$U_{E}=\frac{2\varepsilon \times \zeta \times ƒ\left(\kappa \alpha \right)}{3^{\eta }}$$ (1)

Where U_E_ is the electrophoretic mobility, ε is the permittivity (Farad/m), η is the solution viscosity (mPa.s), κ is the Debye length (nm^-1^) and α is the particle radius (nm). ƒ(κα) is equal to 1.5 based on the Smoluchowski approximation [40].

### Centrifugal stability constant measurement

The stability of the emulsions was evaluated by centrifugal stability constant (Ke) based on the method as reported [41]. Briefly, a certain amount of emulsion was diluted by a factor of 500 with deionized water in a 5 mL tube, and its absorbance value (A) was measured at the wavelength of 490 nm after mixing, using a microplate reader. One 1 ml aliquot of the same emulsion was transferred into a 1.5 ml centrifuge tube and centrifuged at 1520 g for 15 min at 20°C in a high-speed centrifuge. A certain amount of subnatant was diluted by a factor of 500 with deionized water in a 5 mL plastic tube and its absorbance value (A_0_) was measured at the wavelength of 490 nm after mixing. The centrifugal stability constant (Ke) was calculated as follows:

$$K_{e}=\frac{\left(A-A_{0}\right)\times 100\%}{A_{0}}$$ (2)

Where A is the absorbance at 490 nm of the solution before centrifuge, while A_0_ is the absorbance after centrifuge.

### Analysis of lutein stability during storage at different temperatures

The content of lutein was represented by the absorbance measurements (450 nm) detected using a UV–visible spectrophotometer. Before spectrophotometric measurements, the emulsions were diluted 100 times in DMSO that dissolved lutein, oil, and protein to form transparent solutions suitable for UV–visible analyses.

### Statistical analysis

Statistical analyses were carried out using the statistical program SPSS Version 17.0. Comparisons among data of different groups were performed with one-way or two-way ANOVA, where LSD method or Dunnett test were used on the basis of the homogeneity test. Student t test was applied where only two groups’ data were compared. Results were presented as mean ± standard error (SEM) and considered to be significantly different when *p* < 0.05.

## Results

### Effect of whey protein concentration on the particle size and distribution of emulsions

The whey protein concentration affected the particle size of lutein emulsion particles. As for WPI, both low protein concentration (1%) and high protein concentration (10%) increased the particle size and the width of distribution, whereas over 3% of PWP had larger particle size. (see Table 1).

**Table 1:** Effect of different whey protein concentration on the particle size and distribution of emulsions.

concentration	WPI		PWP	
(w/v)	D_z_(nm)	PDI	D_z_(nm)	PDI
1%	219±18^a^	0.33±0.02^a^	209±3.3^a^	0.27±0.02^a^
3%	202±9.7^b^	0.29±0.02^b^	227±6.1^b^	0.30±0.02^a^
5%	208±2.7^ab^	0.26±0.02^b^	228±8.5^b^	0.30±0.02^a^
7%	203±2.5^b^	0.25±0.02^b^	224±7.1^b^	0.26±0.02^a^
10%	213±9.0^a^	0.32±0.03^a^	230±5.0^b^	0.22±0.02^b^

Note: Value = mean ± SEM, n = 3; only values with different lowercase letters were considered significantly different within the same column at p<0.05. Lutein content was 2.5 mM in tested emulsions.

### Effect of lutein content on the particle size and distribution of emulsions

Lutein content had little influence on the particle size but slightly changed the distribution in these two types of whey protein based emulsions. Specifically, 1 mM of lutein showed the narrowest width for both emulsions. The details were shown in Table 2.

**Table 2:** Effect of different lutein concentration on the particle size of emulsions.

Lutein	WPI		PWP	
(mM)	D_z_(nm)	PDI	D_z_(nm)	PDI
0	203±8.0	0. 36±0.03^a^	225±13	0.41±0.03^a^
1	204±2.8	0.30±0.02^b^	228±1.5	0.34±0.02^b^
2.5	209±9.1	0.37±0.03^a^	230±11	0.40±0.04^a^
5	211±8.6	0.35±0.03^a^	227±5.9	0.32±0.03^b^

Note: Value = mean ± SEM, n = 3; only values with different lowercase letters were considered significantly different within the same column at p<0.05. Lutein content was 2.5 mM in tested emulsions.

### Effect of pH on the zeta-potential of the emulsions

The isoelectric point (PI) of beta-lactoglobulin, the main component of whey protein is around 5.2. Consistently, when the pH was distant from the PI, the absolute value of zeta-potential was larger. There was no significant difference between WPI and PWP emulsions at the same pH condition (Table 3).

**Table 3:** Effect of pH on the zeta-potential of the emulsions.

pH	WPI	PWP
2	57.7±3.4	53.6±3.2
3	29.1±2.5	28.3±1.9
4	18.1±0.7	22.8±1.8
6	-45.6±1.9	-43.3±1.5
7	-50.6±3.5	-48.6±2.3
8	-60.6±3.5	-60.7±3.4

Note: Note: Value = mean ± SEM, n = 3. Lutein content was 2.5 mM in tested emulsions. No significant difference was found between WPI and PWP groups.

### Physical stability during storage

**Fig 1:**
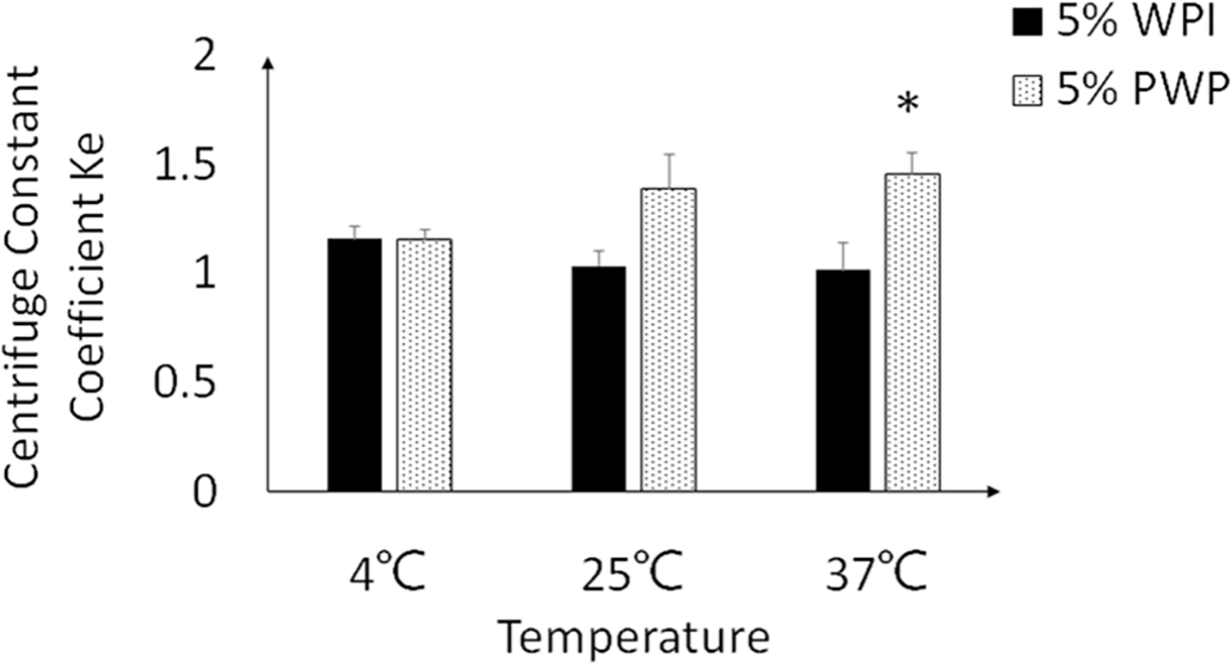
The centrifuge constant coefficient Ke values of the dispersions with WPI or PWP at different temperatures. The centrifuge constant coefficient Ke values of the dispersions with WPI or PWP were tested at temperatures of 4°C, 25°C and 37°C. * means significant different between WPI and PWP based nano-emulsions at p<0.05. Value = mean ± SEM, n = 3.

**Fig 2:**
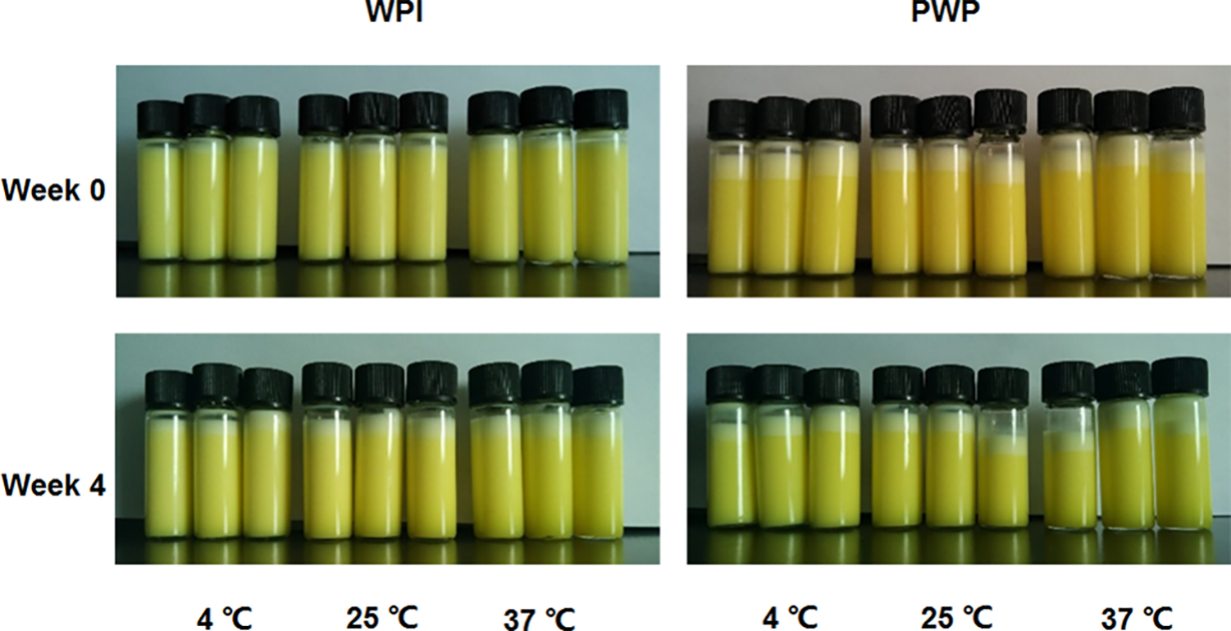
The stability of WPI or PWP based lutein emulsions at different temperatures during storage. PWP showed stratification within one week at all temperatures tested, whereas only WPI based lutein loaded emulsion was stable during four weeks’ storage at 4°C. The WPI emulsion showed a little of stratification at 25°C and 37°C.

After the nano-emulsions were prepared, the centrifuge stabilities of the emulsions were immediately evaluated based on the centrifuge constant coefficient. The PWP based nano-emulsion had significant lower centrifuge stability compared to the WPI based emulsions (Fig 1). Consistently, the PWP emulsion showed stratification just overnight. The WPI based nano-emulsion was stable at 4°C, but showed appearance of stratification at 25°C and 37°C after four weeks’ storage (Fig 2).

### Particle size change of lutein-loaded emulsions during storage

As PWP emulsion was stratified in early stage, we only recorded the particle size change of the WPI based emulsions during four weeks’ storage. The particle size of WPI emulsion began to significantly increase after three weeks’ storage at 25°C and 37°C by approximately 4%, whereas the particle size of the emulsion at 4°C was nearly constant during the experiment (Table 4).

**Table 4:** Storage stability of lutein-loaded emulsions.

Emulsifier type	weeks	storage temperature (°C)		
		4	25	37
WPI	0	209±9.1	209±9.1^a^	209±9.1^a^
	1	214±7.3	217±6.3^ab^	212±6.9^ab^
	2	209±3.6	216±4.1^ab^	218±7.7^ab^
	3	216±5.0	220±4.6^b^	221±5.9^b^
	4	208±1.1	224±6.2^b^	229±7.8^c^

Note: Value = mean ± SEM, n = 3; only values with different lowercase letters were considered significantly different at the same column at p<0.05. Lutein content was 2.5 mM in tested emulsions.

### Lutein retention of lutein-loaded emulsions during storage

**Fig 3:**
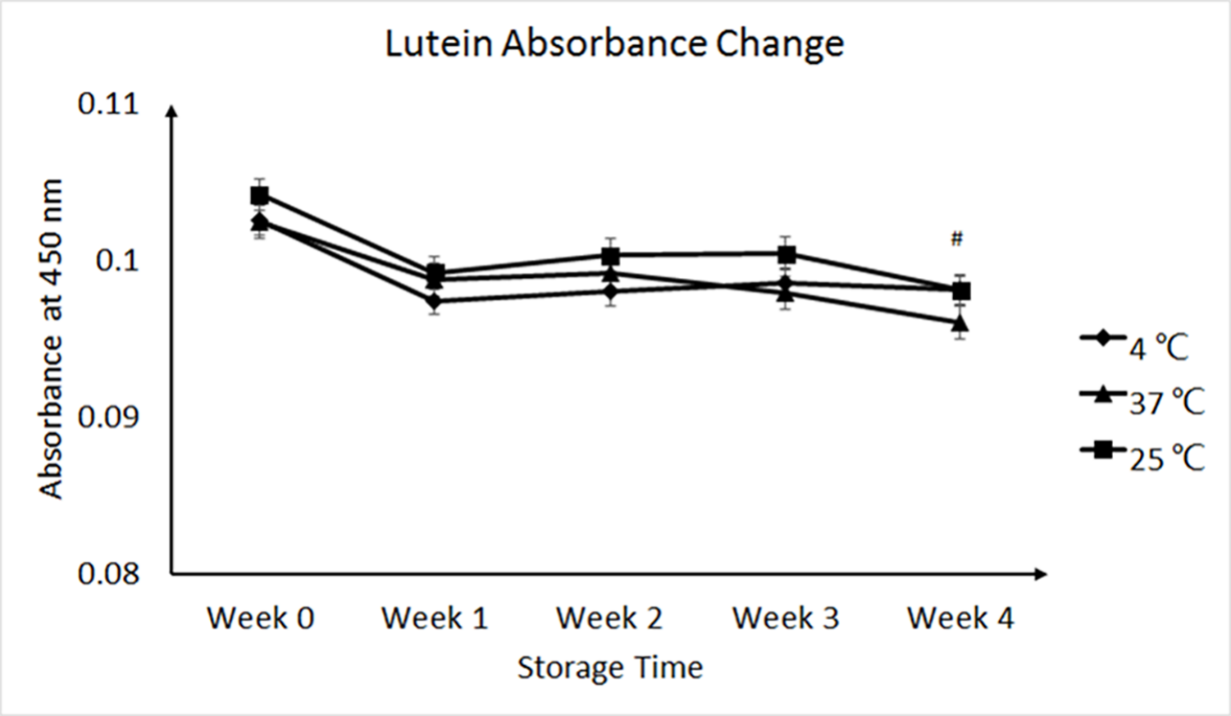
Absorbance change of the dispersions with whey proteins at different temperatures during storage. Absorbance change of the dispersions with whey proteins at temperatures of 4°C, 25°C and 37°C were recorded weekly during storage. ^#^ Significantly different compared with the values at week 0 (p<0.05). No significant change was observed by temperature, though the dispersion showed lower absorbance at 37°C. Value = mean ± SEM, n = 3.

Lutein content was slightly reduced indicated by the decrease of absorbance by approximately 5% after four weeks’ storage, but no significant change of absorbance was observed at different temperatures (Fig 3).

## Discussion

Whey protein is a type of nutritional protein with multiple functions including emulsifying property [42]. As a result, whey protein can be extensively applied in food products, for example, to form emulsions that can increase the stability and bioavailability of hydrophobic molecules [43]. Additionally, whey protein is little digestible by pepsin, but can be hydrolyzed rapidly by proteases such as chymotrypsin and trypsin in intestinal juice, making whey protein to be ideally suitable for controlled release for bioactive compounds [44]. We recently reported that PWP had better emulsifying property than that of WPI at proper ultrasound treatment [32]. Therefore, we tested both in a lutein loaded nano-emulsion system.

Nanotechnology is promising in increasing micronutrient bioavailability [45]. The droplet size of nano-emulsion usually falls typically in the range 20–200 nm [46]. The particle size of the emulsions was all around 200 nm—close to the threshold for toxicity [47], indicating that we successfully generated the food grade nano-emulsions with the assistance of high intensity ultrasound. The emulsions were shown to be stable at pH father than its isoelectric point. This was consistent with previous reports associated with emulsions containing milk protein-coated lipid droplets [26, 48]. Considering the hydrogen ion concentration in common food, we selected pH 7 for preparation of the whey protein solution. Generally, when the relative value of zeta-potential is greater than 30, the dispersion usually has good stability [49]. At pH 7, both nano-emulsions had proper zeta-potentials, indicating their potential to be stable during storage. However, the PWP nano-emulsion with good size and high emulsifying property showed quick appearance of stratification, which was partly predicted by its low centrifuge stability. It’s possible that the inclusion of ethanol in the system interfered the reaction between whey protein and the lutein [50]. Optimization for the emulsion formulation process might solve this problem.

Temperature is an important factor for emulsion stability during storage. High temperature usually leads to increase of frequency in particle collision and promotes aggregation under certain conditions [51]. The WPI based nano-emulsion was stable within four weeks at 4°C, but showed appearance of stratification at 25°C and 37°C. The result was a little different from the report of Frede *et*. *al*. [25]. Our result was better than theirs in particle size, which was probably attributable to the use of high intensity of ultrasound. However, they used β-lactoglobulin and whey protein hydrolysate instead, which was possibly the cause for the difference. Another possibility was the inclusion of ethanol in the system that affected the emulsions in a concentration dependent way [50].

Lutein content was slightly reduced indicated by the decrease of UV absorbance by approximately 5% after four weeks’ storage, which was probably because of the occasional light exposure—a factor that easily degrade lutein [52]. Lutein was abruptly reduced after one week and then kept at a constant level, which was probably attributed to loss of little amount of free lutein that failed to be effectively encapsulated.

From above, we conclude that the WPI based lutein loaded nano-emulsion as in the Oil/Water/Ethanol system prepared with assistance of high intensity of ultrasound has high potential of improving the stability of lutein during storage, which is promising for future use as a good carrier system for hydrophobic bioactive molecules.
